# Supplementary material for: USP7 Deregulation Impairs S Phase Specific DNA Repair after Irradiation in Breast Cancer Cells
Source: Biomedicines. 2024 Mar 29;12(4):762. doi: 10.3390/biomedicines12040762 (PMC11047985; doi:10.3390/biomedicines12040762)
Supplement: Supplementary file 1 [file biomedicines-12-00762-s001.zip › biomedicines-2896802-supplementary.pdf]

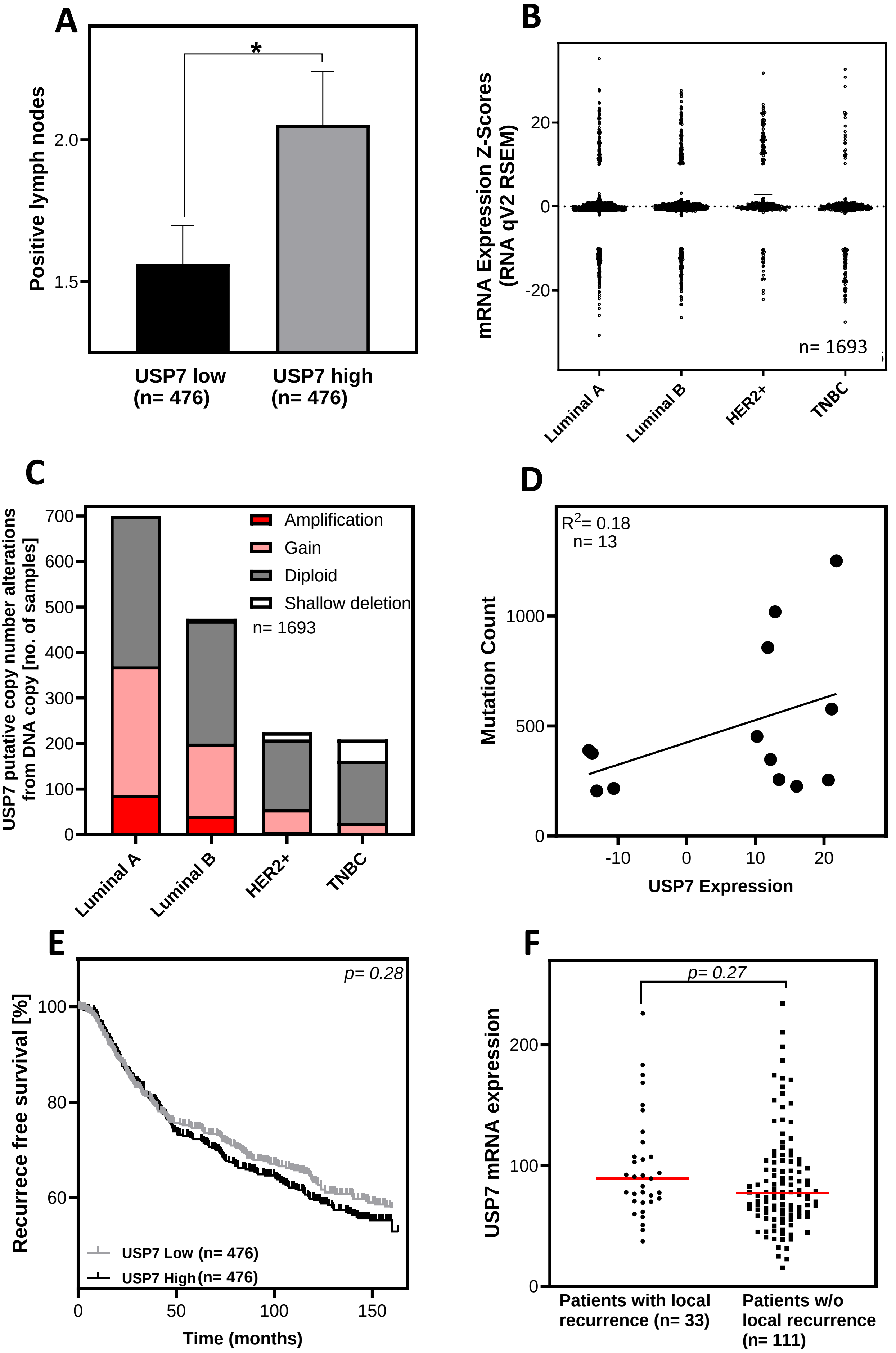

Suppl. Fig. S1: Effects of USP7 overexpression in breast tumors and breast cancer cell lines. A: Positive lymph nodes in the extreme quartiles of USP7 mRNA expressing breast tumors. Data were extracted from the METABRIC dataset. **B**: mRNA expression of USP7 in four breast cancer subtypes. Data were extracted from the METABRIC dataset, and sorted by the molecular subtype (n= 1693). **C**: DNA copy number alterations in breast tumors of the four main molecular subtypes (n= 1693 out of 1904 samples). Data were extracted from the METABRIC dataset and sorted by the molecular subtype. **D:** Mutation count in USP7 high expressing and low expressing breast cancer cell lines. Data were extracted from the Cancer Cell Line Encyclopedia dataset. E: Recurrence free survival in the extreme quartiles of USP7 mRNA expressing breat tumors. Data were extracted from the METABRIC dataset. F: USP7 expression in patients with local recurrence and patients without (w/o) local recurrence. Red lines indicate mean values. Data were extracted from the Metastatic Breast Cancer Project dataset. Asterisks represent significant differences (*p < 0.05, Student's t-test).

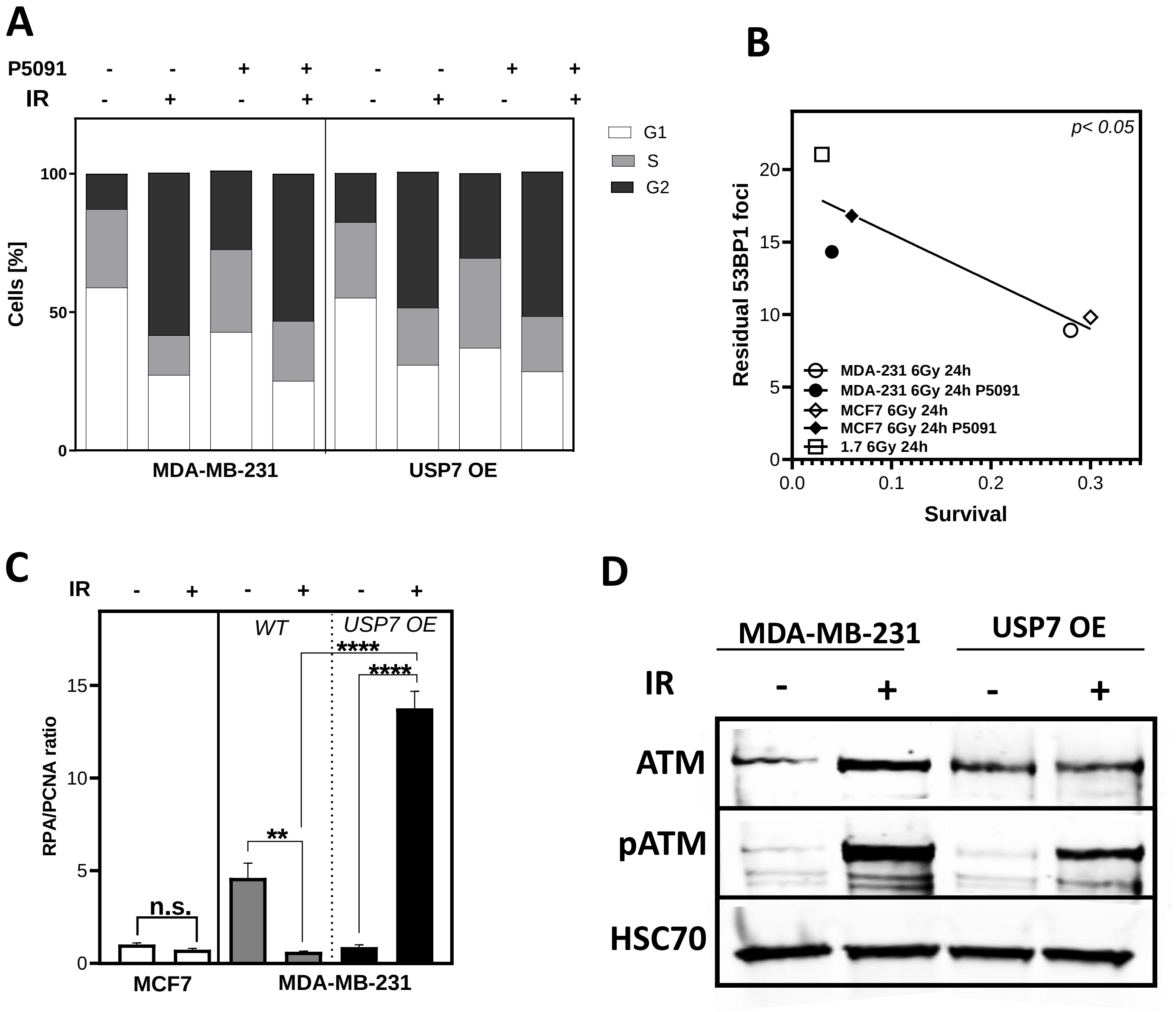

Suppl. Fig. S2: Effects of UPS7 overexpression and inhibition in breast cancer cell lines. A: Cell cycle distribution in MDA-MB-231 and isogenic cells with USP7 overexpression (USP7 OE). Percentage of cells 6 h after 6 Gy and/or P5091 treatment (2 µM). **B**: Correlation of residual 53BP1 foci and cellular survival after irradiation. **C**: Ratio of PCNA and RPA foci without or 6 h after 6 Gy irradiation. **D**: Representitive image of Western blot immunodetection of ATM/pATM in MDA-MB-231 cells and isogenic USP7 OE cells. HSC70 served as loading control.
